# Current-induced mechanical torque in chiral molecular rotors

**DOI:** 10.3762/bjnano.14.57

**Published:** 2023-06-12

**Authors:** Richard Korytár, Ferdinand Evers

**Affiliations:** 1 Department of Condensed Matter Physics, Faculty of Mathematics and Physics, Charles University, Ke Karlovu 5, 12116 Praha 2, Czech Republic; 2 Institute of Theoretical Physics, University of Regensburg, D-93050 Regensburg, Germany

**Keywords:** molecular junctions, molecular motors, molecular switches

## Abstract

There has been great endeavor to engineer molecular rotors operated by an electrical current. A frequently met operation principle is the transfer of angular momentum taken from the incident flux. In this paper, we present an alternative driving agent that works also in situations where angular momentum of the incoming flux is conserved. This situation arises typically with molecular rotors that exhibit an easy axis of rotation. For quantitative analysis we investigate here a classical model where molecule and wires are represented by a rigid curved path. We demonstrate that in the presence of chirality, the rotor generically undergoes a directed motion, provided that the incident current exceeds a threshold value. Above this threshold, the corresponding rotation frequency (per incoming particle current) for helical geometries turns out to be 2π*m*/*M*_1_, where *m*/*M*_1_ is the ratio of the mass of an incident charge carrier and the mass of the helix per winding number.

## Introduction

Experiments employing scanning tunneling microscopy (STM) have achieved the directed rotation of molecules controlled by an electrical current. Correspondingly, realizations of molecular switches and rotors have been reported, [1–9], with potential relevance for future molecular technologies.

The theory describing the working principle of such molecular motors often employs angular Langevin equations [9–10]. This method has been established by Hänggi [11] and Astumian [12] and their collaborators in the context of Brownian motors. It describes the dynamics of a classical angular variable ϑ that is subject to a “ratchet”-type potential in the presence of a (phenomenologically treated) driving torque. Ab initio expressions for the current-induced torques have been obtained within the non-equilibrium Green’s function formalism [13–14]. The current excites a variety of molecular vibrational modes, rendering the atomistic analysis of the torque very complex (see [6] for an ab initio calculation of the vibrations).

To bring about a controlled unidirectional rotation in the STM setup requires a degree of symmetry breaking. There are two typical situations, that is, either the molecule by itself exhibits a handedness (chirality) or chirality is imposed by the geometry of the molecular junction [6,10]. The purpose of this article is to provide a qualitative description of the current-induced mechanical torque within a toy model framework.

**Figure 1:**
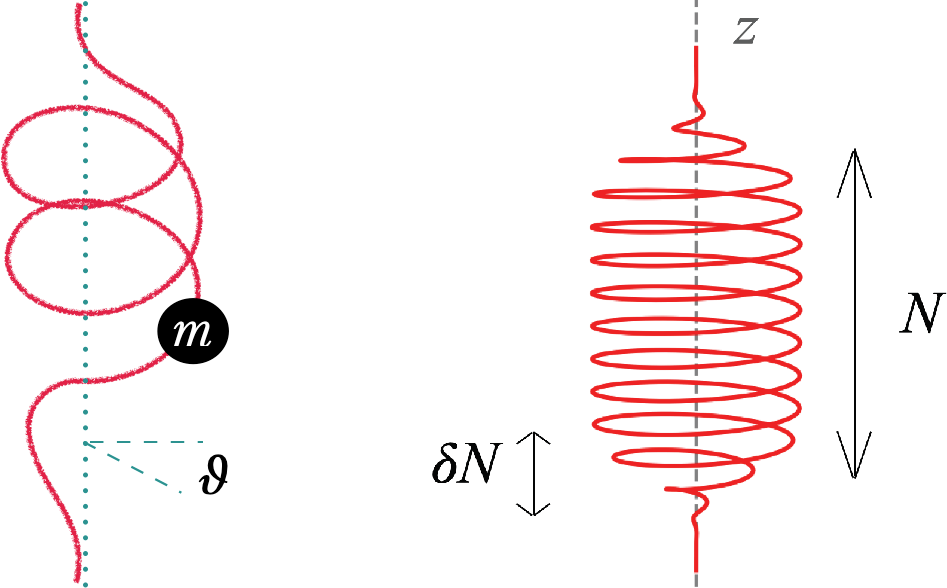
Left: A particle of mass *m* is constrained to move along a path (red curve). An axis is assumed so that the initial and final radius of the path with respect to the axis is zero. Furthermore, the path is allowed to rotate (angle ϑ) around the axis. Right: An *N*-helix that smoothly evolves from a straight line to its radius *R*, parameterized by the formulas in the Appendix section. Here, the number of turns is *N* = 10 and the full radius sets in from zero after δ*N* = 1 turns.

We consider a classical model of the molecular rotor where the molecule is modeled as a one-dimensional curve (“molecular wire”) that guides the flow of the charge carriers (see Figure 1 (left) for illustration). The motion of the particle along the molecule obeys Lagrangian dynamics. The wire can rotate around a given axis with angle ϑ. The torque driving the rotation is provided by the back action of moving particle. In the absence of a potential *V*(ϑ), angular momentum is conserved.

The main outcome of this work is that the wire rotates even if the net transfer of angular momentum of the transmitted particles is zero. The operation principle is that the particle exerts a torque when entering and leaving the molecular wire. Even if both exactly compensate, the wire rotates while the particle travels along, so that each transmitted particle results in a shift δϑ. This operational principle is different than an earlier reported one [15], where an electric field was needed to continuously accelerate the electrons while they travel along a helical wire. When the rotation of the molecule is hindered by a potential barrier *V*(ϑ), we find that the mass current needs to overcome a threshold for the wire to rotate. The resulting time-averaged angular velocity is time-independent and directional for all supercritical currents.

Finally, we consider a situation where the net torque exerted by the transmitted particle does not vanish. In this situation, the rotation trivially appears due to the angular momentum transfer (“garden hose effect”). This situation represents molecular junctions where the incoming or outgoing current can carry a non-vanishing angular momentum. We present the characteristics of the crossover between both regimes.

To exemplify our results, we employ a helical geometry. Helical molecular wires have sparked a lot of attention because of reports of spin-selective transport [16–18]. This phenomenon falls under the umbrella term “chirality-induced spin selectivity (CISS)”. The full explanation of CISS remains elusive, however [19]. Therefore, the problem of current-induced angular momentum generation in helical molecules remains open, with broader scientific and technological significance.

Summarizing, our work provides insights into the operation principles of molecular rotors, specifically the velocity–current characteristics and threshold currents. Our results can support the design of nanoscale mechanical devices.

## Model

### Model geometry (kinematics)

Our classical model contains a particle (mass *m*) moving on a rigid path, which can rotate around an axis, see left part of Figure 1. The rotation angle of the path is denoted by ϑ. In absence of a rotational degree of freedom of the path, the particle would experience constrained dynamics. With the rotation allowed, the motion of the particle can exert a torque on the path. Conversely, the dynamics of the path around its angle affects the passage of the particle.

The trajectory (path) at rest (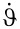 = 0, ϑ = 0) will be expressed parametrically in a cylindrical coordinate system:


[1]

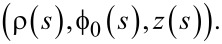



The parameter *s* could be the distance along the path; for the purpose of this work it is not required. For simplicity, we further stipulate that *z*(*s*) is monotonously increasing with *s*, and that the trajectory never intersects itself.

The model contains two dynamical variables, the degree of freedom of the particle, *s*(*t*), and ϑ(*t*), the latter being the angle of the path with respect to a static coordinate system. Our aim is to investigate the dynamics of ϑ under the condition that the incoming and outgoing particles do not carry any angular momentum. We achieve this by conditioning the path to have vanishing radius at its start and at the end,


[2]

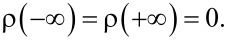



Later on, we also employ a path with a finite final radius, allowing for an angular momentum transfer. As we shall demonstrate, paths satisfying Equation 2 will still turn when subjected to particles, if the path is chiral (lacks reflection symmetry). We shall employ a helical path, with the radius smoothly raising from zero, effecting *N* turns, and decreasing at the end, see Figure 1b. The mathematical expression of the path can be found in the Appendix section.

### Lagrangian dynamics

We construct the equations of motion from a Lagrangian, 
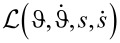
.

#### Static path

For a fixed path, ϑ = 0, the Lagrangian of this model reduces to the Lagrangian of a particle subject to a constraint,


[3]

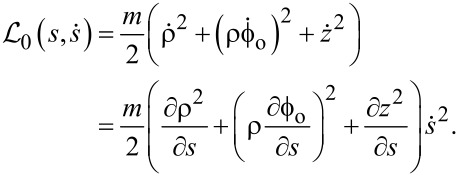



Formally, as a consequence of the constraint, the model adopts the form of a free particle with an *s*-dependent mass. Recalling the conservation of energy, the formal integration of the Lagrangian in Equation 3 is trivial.

#### Dynamic path

To allow for a dynamical rotational degree of freedom ϑ for the path, we now introduce the actual angle ϕ of the particle in a static cylindrical system, defined by


[4]

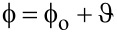



and introduce it in Equation 3.

Without a particle on a path, the dynamics of the rotor will be governed by the kinetic energy 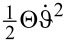 and the potential energy *V*(ϑ).

The full Lagrangian of the coupled system becomes


[5]

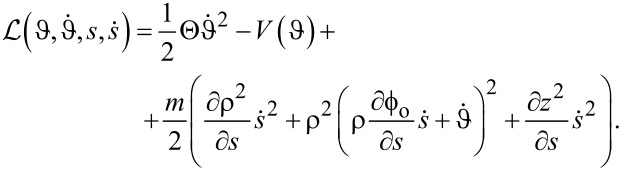



It provides two equations of motion (EOM), which are listed in the Appendix section. We integrate the EOM using a Runge–Kutta method, see Appendix section.

#### Basic parameters and scales

The parameters that enter the coupled dynamical problem governed by the Lagrangian in Equation 5 are: (1) The particle mass *m*. (2) The definition of the path (Equation 1); it will be assumed that the path has a characteristic radius *R* ∼ maxρ(*s*), which will serve as a length scale. Helical paths are primarily distinguished by the number of turns *N*, which controls the time particle spends on the helix. (3) The moment of inertia of the path Θ; the latter can be expressed through the characteristic radius as *MR*^2^, defining mass *M*. The ratio μ = *m*/*M* enters in the collision characteristics. (4) The potential *V*(ϑ) that hinders the motion of the path (setting a preferred direction). The difference between minimum and maximum is denoted by Δ*V*. This is the energy scale that needs to be overcome when inducing an unbound rotation; if not, one is trapped in the potential valley. (5) The above parameters of the path combine to give a time scale 
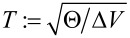
, which equals 0.334 times the period of small harmonic oscillations of the path without the particle around the potential minimum. We shall use *T* as a unit of time in our numerical results. (6) The initial velocity of the particle, at *s* = −∞, denoted by 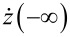, that is “the impact velocity”. A suitable unit for the latter is *R*/*T*, and it is inversely proportional to the time spent in the curved path. The precise initial placement of the particle, *z*(−∞), is irrelevant because the particle decouples from the path when ρ = 0.

### Conservation laws

Consider a single collision event, with particle starting at *s* = −∞, passing through the rotor (where ρ ≠ 0) and leaving towards *s* = ∞.

#### Energy conservation

If before the collision the path is at rest, energy conservation implies that


[6]

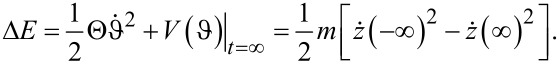



This is a consequence of the invariance of the Lagrangian in Equation 5 with respect to time translations. The right hand side of Equation 6 describes the energy loss of the particle after the collision. We shall focus on the regime where the energy gain of the path, Δ*E*, is small, usually not higher than Δ*V*. In the limit of fast impact velocities, Equation 6 implies that the relative decrease of the particle velocity after the collision is small.

#### Angular momentum conservation

When *V*(ϑ) = const., the Lagrangian in Equation 5 is invariant with respect to rotations. The total angular momentum


[7]

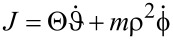



is time-independent. For paths satisfying Equation 2, *J* equals the angular momentum of the path before and after the collision.

### Path under a current: stroboscopic dynamics

We will also investigate a dynamics of the path when the particles appear sequentially, that is, the path under a current. When the particles arrive to the path periodically, with period Δ*t*, the particle current reads


[8]

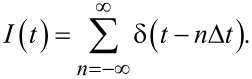



The time-averaged current reads ⟨*I*(*t*)⟩ = *I* = 1/Δ*t*.

We will assume for each incident particle identical initial conditions, that is, at each time *n*Δ*t* the same *z* and 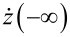. However, the initial conditions for ϑ and 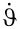 will be different, corresponding to the dynamical state of the path. In between the sequential collisions, the path evolves under its independent equation of motion, 
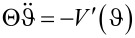
.

We remark that our formalism does not allow more than one particle on the molecule at any time. This approximation is valid in the limit of low currents. Even in this limit, we observe an interesting threshold behavior.

## Results

First, we demonstrate how a particle that does not carry any angular momentum can turn the path. It is instructive to begin with the limit of full rotational invariance, when *V* = 0 (section “Rotational invariance”), because conservation laws allow for a straightforward integration of the EOM. Next, we treat analytically the case *V* ≠ 0 in the limit of fast projectiles in the so-called sudden approximation (SA) in section “Broken rotational invariance: analytic considerations in the sudden approximation”. We use the analytical considerations as guiding principles for the analysis of the numerical results in section “Numerical results for single-projectile dynamics”. After that, we consider paths which allow for a finite angular momentum transfer in section “Directed motion of a helix with an open end”.

### Rotational invariance

When *V* = 0, the dynamics of a single shot (collision) is entirely captured by angular momentum conservation. Let us assume the path at rest before the collision. Equation 7 with *J* = 0 binds the change of the angle of the particle with the change of the angle of the path (analogous to Keppler’s law)


[9]

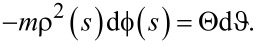



Integrating from *s* = −∞ to *s* = ∞, we obtain


[10]

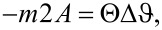



where, on the left-hand side, 
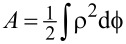
 denotes the area described by the “clock” with a variable radius ρ(*s*) during the passage of the particle. For the *N*-helix, *A* ≈ π*R*^2^*N*. On the right-hand side, we obtain the change of the angle of the path.

Although the path can experience a turn, no angular velocity is generated after the collision, as a consequence of Equation 2. The traversing particle does exert a torque. However, when the torque is integrated over time, it produces no net angular velocity. The path experiences a turn in a preferred direction. For a general path, the turn is finite, if the “clock” area is finite. This situation can not be realized in paths with a spatial reflection plane or an inversion point located on the path. For paths with handedness (chirality), the sign of the turn is determined by the sign of *A*, which, in turn, has the chirality sign.

Next, while still assuming *V* = 0, we ponder three specific sectors centered around ϑ = 0, ±2π/3 and investigate the conditions for a single particle to switch the *N*-helix from one sector to another. The condition is that Δϑ exceeds ±2π/6. Combining with Equation 10, we arrive at


[11]

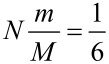



(or more precisely, 
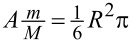
). In the above formula, *M* is proportional to *N*, so that the required threshold particle mass *m* is independent on the length of the helix.

### Broken rotational invariance: analytic considerations in the sudden approximation

We will be concerned with a situation in which the rotation of the path is hindered, as in the experimental realizations of axial molecular switches. In such situations, the hindering potential *V*(ϑ) should have at least three minima in order to discretize a directed circular motion. Therefore, we choose


[12]

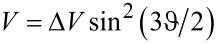



with three minima (preferred directions), separated by obstacles of the height Δ*V*. Experiments on three-state rotors have been reported recently [6,10]. Rotational invariance is broken and although there is energy conservation, the equations of motion are difficult to treat analytically. However, there is a limit in which approximations are feasible.

#### Single particle dynamics

If the passage time of the particle δ*t* is much smaller than the oscillation period 
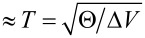
, we may safely neglect the potential in the collision problem. The formulas in Equation 10 and Equation 11 remain valid in this limit and will serve us as a useful guide. The abovementioned condition for the applicability of the formulas of the sudden approximation can be formulated as


[13]

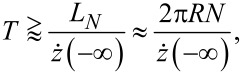



where the passage time on the right-hand side has been approximated from a uniform motion of the projectile over the path length *L_N_* (where ρ *>* 0). For an *N*-helix parameterized in the Appendix section,


[14]

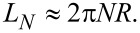



An important feature in the broken rotational invariance is that the restoring torque gives the path an acceleration once the particle disappears. The resulting motion can be, in general, bound to the potential minimum or unbound, depending on the parameters. In the SA, the condition that separates the two regimes is expressed by Equation 11.

#### Helix under a current

A single particle may not cause a turn that is sufficient for an unbound motion if the mass ratio μ = *m*/*M* is too low, for example. But the required critical turn can be effected if particles arrive sequentially, that is, under a current *I*. According to the Equation 11, each particle induces an angular boost as it passes.

If the current runs for a time *t* (to be specified later), Equation 11 becomes


[15]

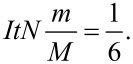



It determines the minimum threshold mass current *I_m_* = *mI* required to perform a switch. The last formula is applicable under the following specific conditions: (1) Equation 16 comes from the SA, demanding that the impact velocity is large enough (see Equation 13). (2) The time between collisions should be much smaller than *T* in order to silence the restoring torque: *I*^−1^ ≪ *T*. (3) The time required to overcome the potential barrier must also be much shorter than *T*, else the path likely performs an oscillatory motion against a displaced minimum.

Our objective will be to determine the threshold mass current *I_m_*. Therefore, the condition in Equation 15 from the SA can be written as


[16]

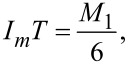



where 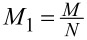 is the mass of a single helix turn. (A more general version replaces *N* by *A*/π*R*^2^.) It should be added that *T* is a function of length because it depends on the mass. The criterion in Equation 16 along its range of validity will be demonstrated numerically in the following section.

### Numerical results for single-projectile dynamics

After the analytic considerations, we resort to the numerical solution of the EOM in order to investigate the situation in which the rotational invariance is broken by the potential, Equation 12, which sets three preferred directions. First, we inspect the applicability of the SA and, then, we investigate the hindered helix subject to the current.

#### Limits of the sudden approximation

**Figure 2:**
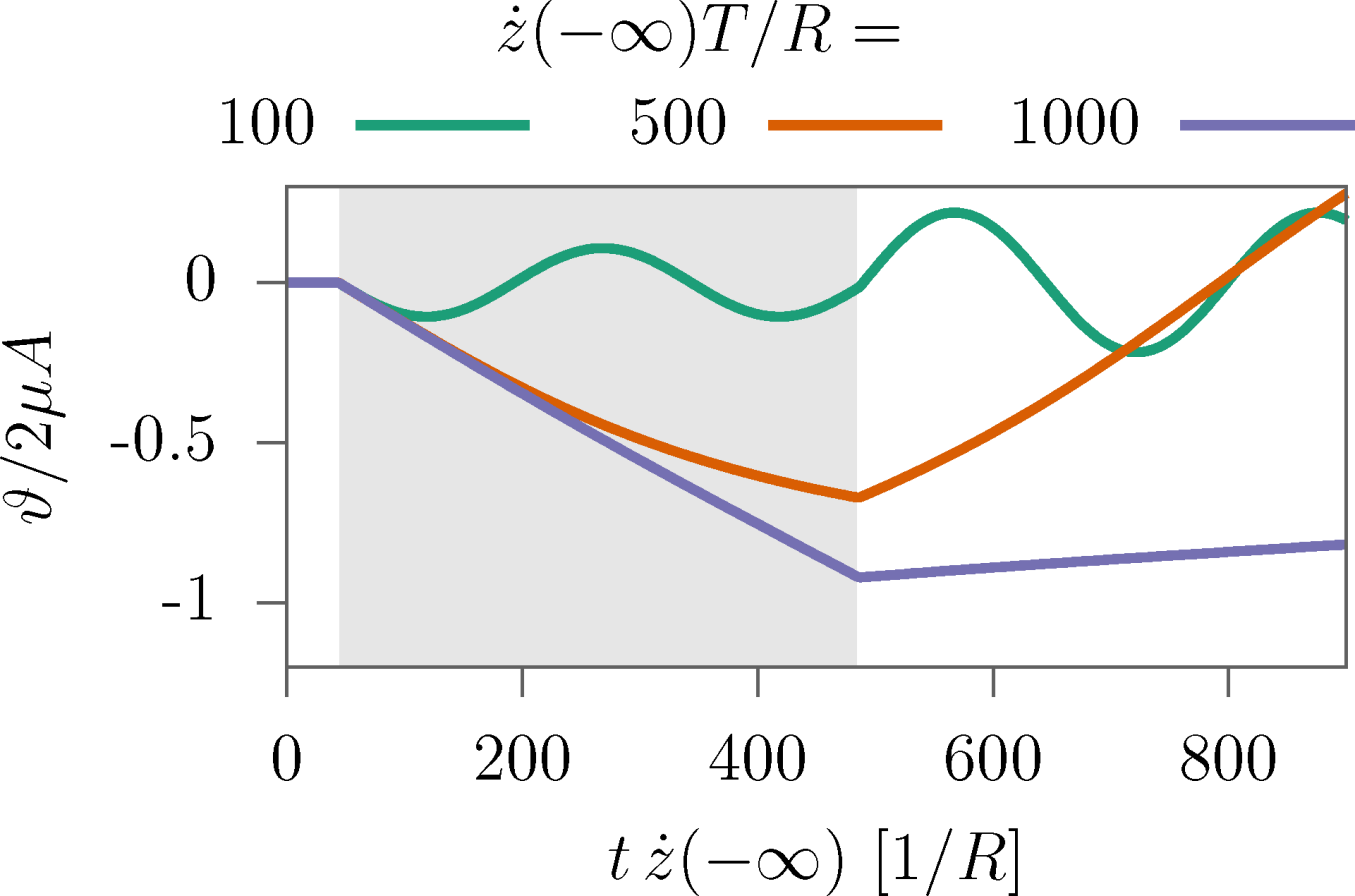
Impact of the passage of a single particle on the angle of a 50-helix. The shaded region denotes the time interval when ρ is nonzero, that is, the particle is in the helix. The traces are parameterized by the impact velocity. The time is rescaled by the velocity in order to make the traces match. The angle is rescaled by factors from the Equation 10, whence −1 indicates the angle in the sudden limit. Other parameters are μ = *m*/*M* = 0.004, δ*N* = 1, and the potential of Equation 12.

Figure 2 shows the time evolution of ϑ during and after a collision with a particle for three different impact velocities. The initial condition for the helix was 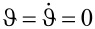 and the particle was put at the entrance of the helix with impact velocity 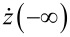.

At the beginning of the collision, for very short times, the three curves lie on top of each other. This is because at these times the restoring torque −*V*′(ϑ) is not very effective. The condition that the passage time is much smaller than *T* is fulfilled at the beginning for all traces. For 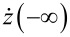
*T*/*R* = 1000, the helix turns with an almost constant velocity when the particle is present, and the total angle reaches the value from the SA. For 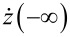
*T*/*R* = 500, the restoring torque markedly bends the curve towards the potential minimum. The slowest projectile (green curve) gives the helix only a small initial velocity. When the projectile is in the body of the helix, the helix oscillates. Here, SA is not applicable, except for very short times. The collapse of the SA predicted by Equation 13 and Equation 14 is 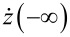
*T*/*R* ≈ 314, consistent with Figure 2.

After the collision, the helix performs either a bound oscillatory motion around the potential minimum or its motion is unbound. In Figure 9 in the Appendix section, we have plotted the critical parameters *m*/*M*, 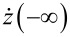, and *N*.

### Helix under a current

The threshold ratio *m*/*M*_1_ = 1/6 is too high to be achieved in molecules in a STM. However, we can make it more favorable if we consider the helix under a particle current *I*, as Equation 16 suggests.

**Figure 3:**
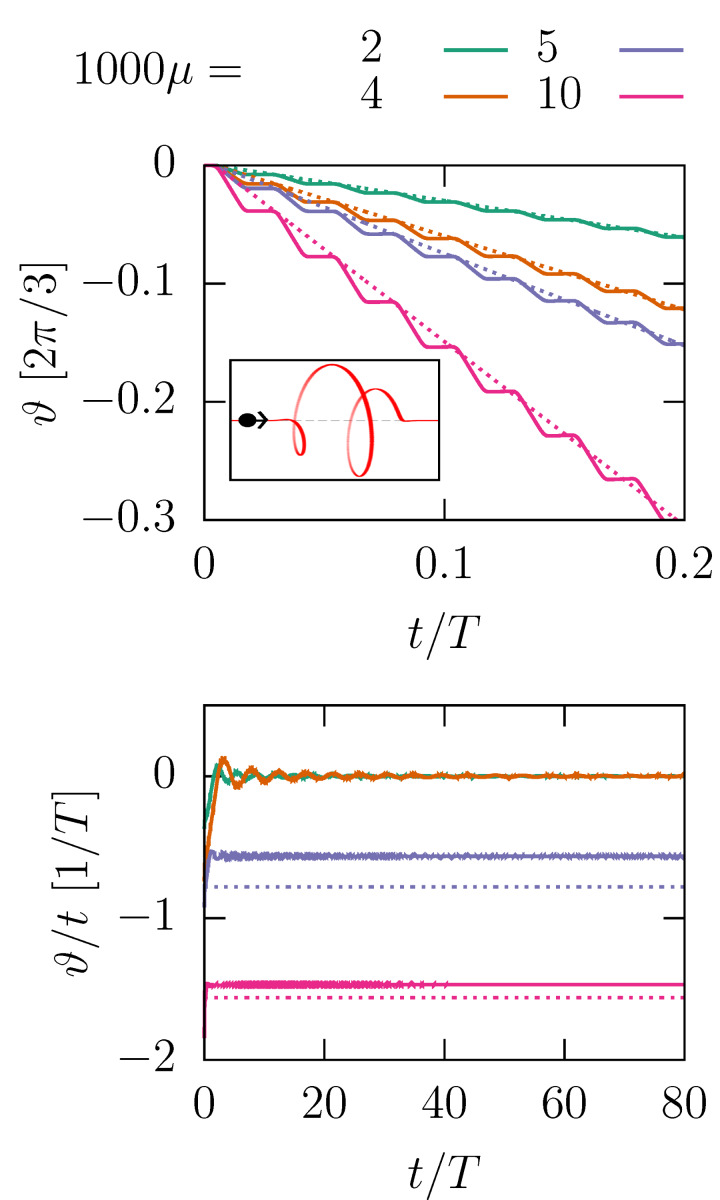
Time evolution of the angle of the path ϑ under a current *IT* = 40, for different mass ratios μ = *m*/*M* and 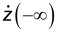
*T*/*R* = 1000. The path is an *N*-helix with *N* = 1.5, δ*N* = 1 (depicted in the inset). Numerical results (solid lines) are complemented by linear evolution from the SA (dotted lines). The steep parts of the saw-tooth profile, visible in the top panel, are in the intervals when the particle moves through the helix. The bottom panel presents ϑ/*t* for long times, showing directed motion for sufficiently large μ.

Figure 3 shows the evolution of the angle when the 1.5-helix is under a current. The traces contain tiny sequential steps, which are more pronounced for large μ. These are the angular boosts produced by the collisions. The plot also shows a comparison with a straight line obtained from the SA, that is, ϑ_SA_(*t*) = −*tI_m_*2*A*/Θ (smoothed over time). The deviation is caused by the restoring torque −*V*′(ϑ), which counteracts the boosts. This countereffect can result in a bound (oscillatory) motion or an unbound directed motion with a constant average angular velocity 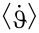 and a small oscillatory component.

How does 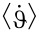 depend on the current? Equation 16 suggest a critical behavior as *I_m_* increases. For large currents, the kinetic energy of the rotor is large compared to Δ*V*; the angular velocity is entirely determined by regular angular boosts of the form of Equation 10, namely,


[17]

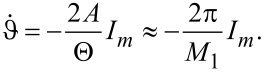



**Figure 4:**
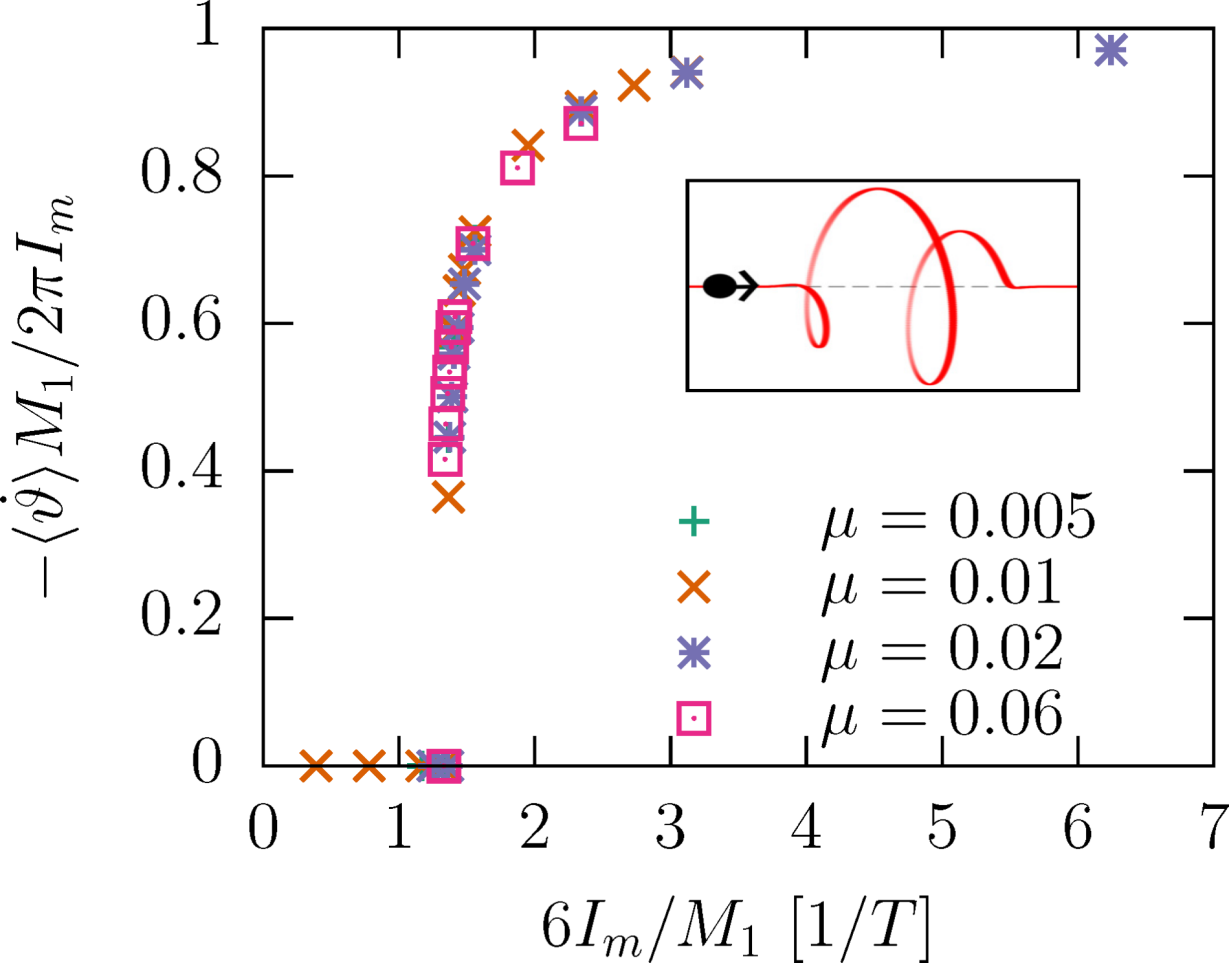
Dependence of the average angular velocity of the helix under a mass current *I_m_* = *mI* for different mass ratios μ = *m*/*M*. *M*_1_ is the mass of a single helix turn; the impact velocity is 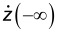
*T*/*R* = 1000. The inset shows that upon entering of the particle, the helix starts revolving counterclockwise when seen from the opposite helix end. Therefore, the helix turns clockwise to compensate the angular momentum. The current therefore causes a constant negative 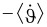.

Figure 4 shows the dependence of the velocity on the mass current for different mass ratios in the fast impact limit. The data points collapse on a single universal curve.

This plot fully encapsulates the mass dependence. It also has a length dependence. As a function of the length, only *T* is expected to change via the linear increase of *M* = *M*_1_*N*. Provided the impact velocity is fast enough, the universal curve has a negligible velocity dependence. Figure 8 in the Appendix section shows that for smaller velocities, the threshold *I_m_* shifts to higher values. The limit Δ*V* → 0^+^ is also of interest: It implies *T* → ∞, and, thus, a vanishing threshold *I_m_*.

### Directed motion of a helix with an open end

#### Angular momentum transfer

In a scanning tunneling setup, the condition in Equation 2 is not always fulfilled, for example, when the tip of the microscope does not bind to the molecule. In our theoretical framework, this situation is represented by a path parameterized by *s* ∈ (−∞, *s*_F_). At the initial point ρ(*s* = −∞) = 0, but at the final point ρ(*s*_F_) := *s*_F_ *>* 0. Thus, as the particle leaves the path at *s*_F_, it transfers angular momentum to the path, see Equation 7. Consequently, the collision causes a boost both in ϑ and 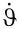.

As long as the restoring torque can be neglected, in the SA we can obtain the angular momentum boost by combining energy and angular momentum conservation laws,


[18]

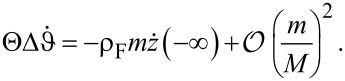



The first term assumes that the velocity of the outgoing particle equals the impact velocity. This velocity must be corrected due to energy transfer, which yields a term of the second order in μ.

#### Switching in the SA

The condition for switching is that the energy gain of the path, Equation 6, must overcome the potential barrier. In the limit of large velocities, the kinetic term (due to angular momentum boosts) dominates over the potential gain via angular boosts, and the condition becomes 
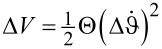
. For a single particle, the switching condition reads 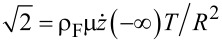.

Under a current, the velocity boosts can be added sequentially, and the condition becomes


[19]

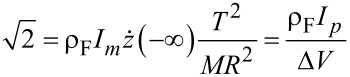



where we introduced the incident momentum current 
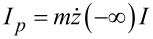
. The nominator of the fraction on the right-hand side can be interpreted approximately as the outgoing angular momentum current, in view of the expansion in Equation 18.

**Figure 5:**
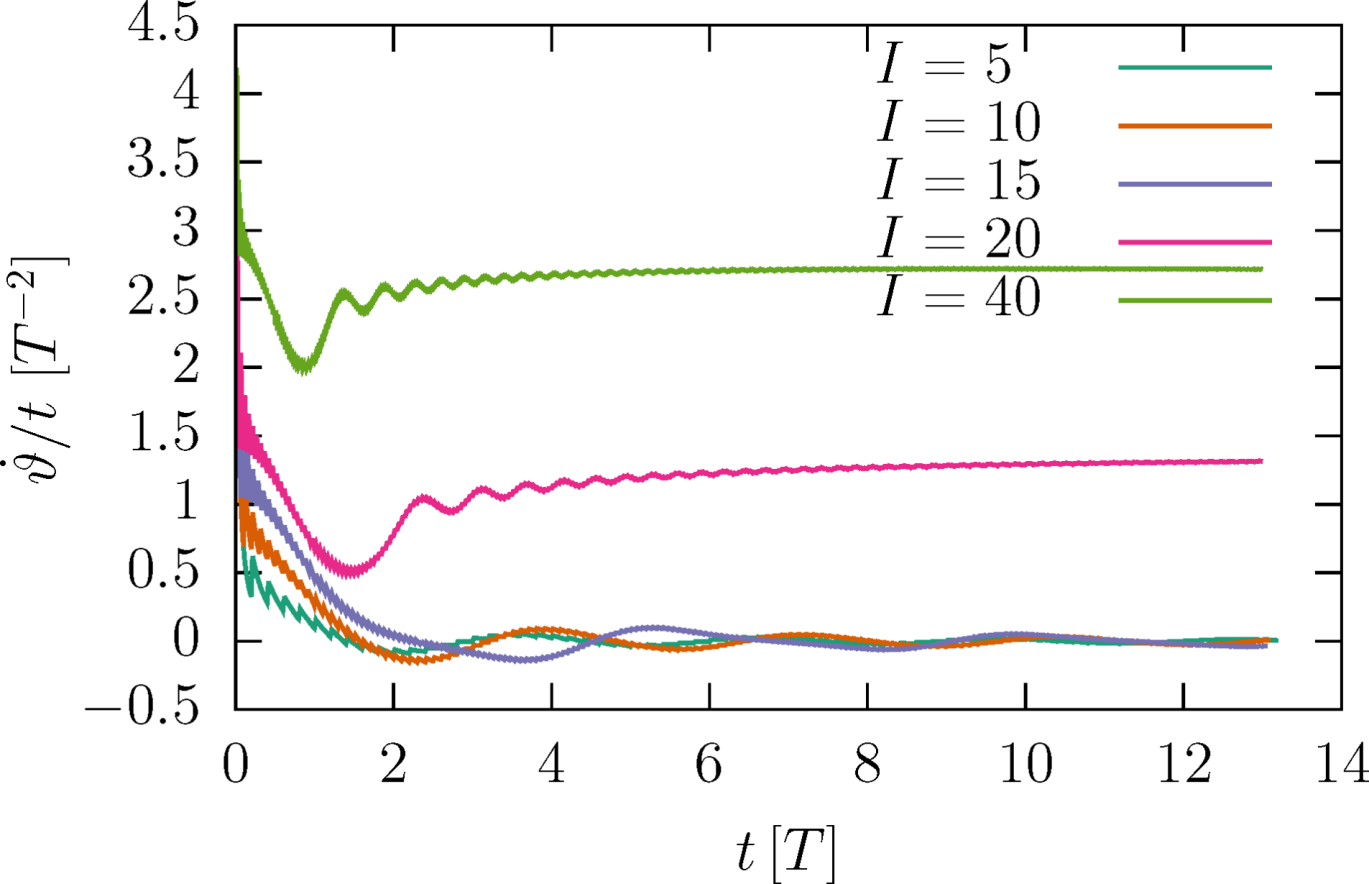
Time evolution of the angular velocity for a path with ρ_F_ = *R*, where the angular momentum boosts dominate. As a function of μ, there is a transition from a bound motion to an unbound motion, with a non-constant velocity.

Numerical simulations confirm the threshold behavior, see Figure 5. Below the threshold, the path is bound to the potential minimum. Above the transition, the path is accelerated, possibly non-uniformly.

**Figure 6:**
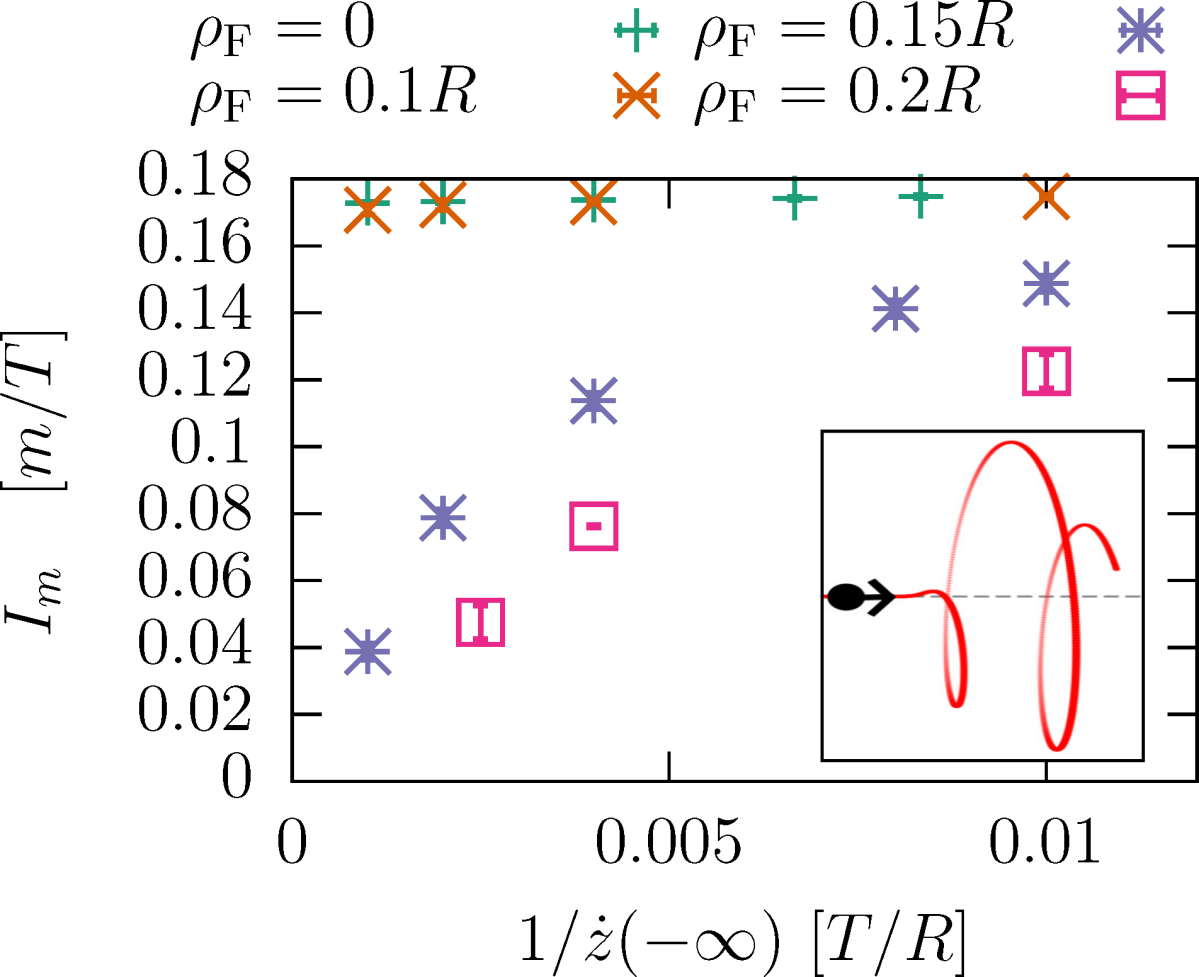
Threshold mass current as a function of the impact velocity for different values of the exit radius ρ_F_. μ = 0.05.

In the next step, we investigate the dependence of the threshold current. For a fixed mass ratio, Figure 6 shows the threshold mass current. There are two regimes covered in that plot: (1) ρ_F_ = 0: the helix is not accelerated. The threshold *I_m_* depends on the impact velocity very weakly in the given range. Actually, it increases with decreasing impact velocity (see Figure 8. This is the mechanism of angular boosts studied in the previous section. (2) For nonzero ρ_F_, the collision causes a net torque, the helix always accelerates. In the limit of large impact velocities the switching due to angular momentum boosts overtakes and the threshold *I_m_* drops inversely proportional to 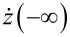. This regime is the familiar garden hose effect.

**Figure 7:**
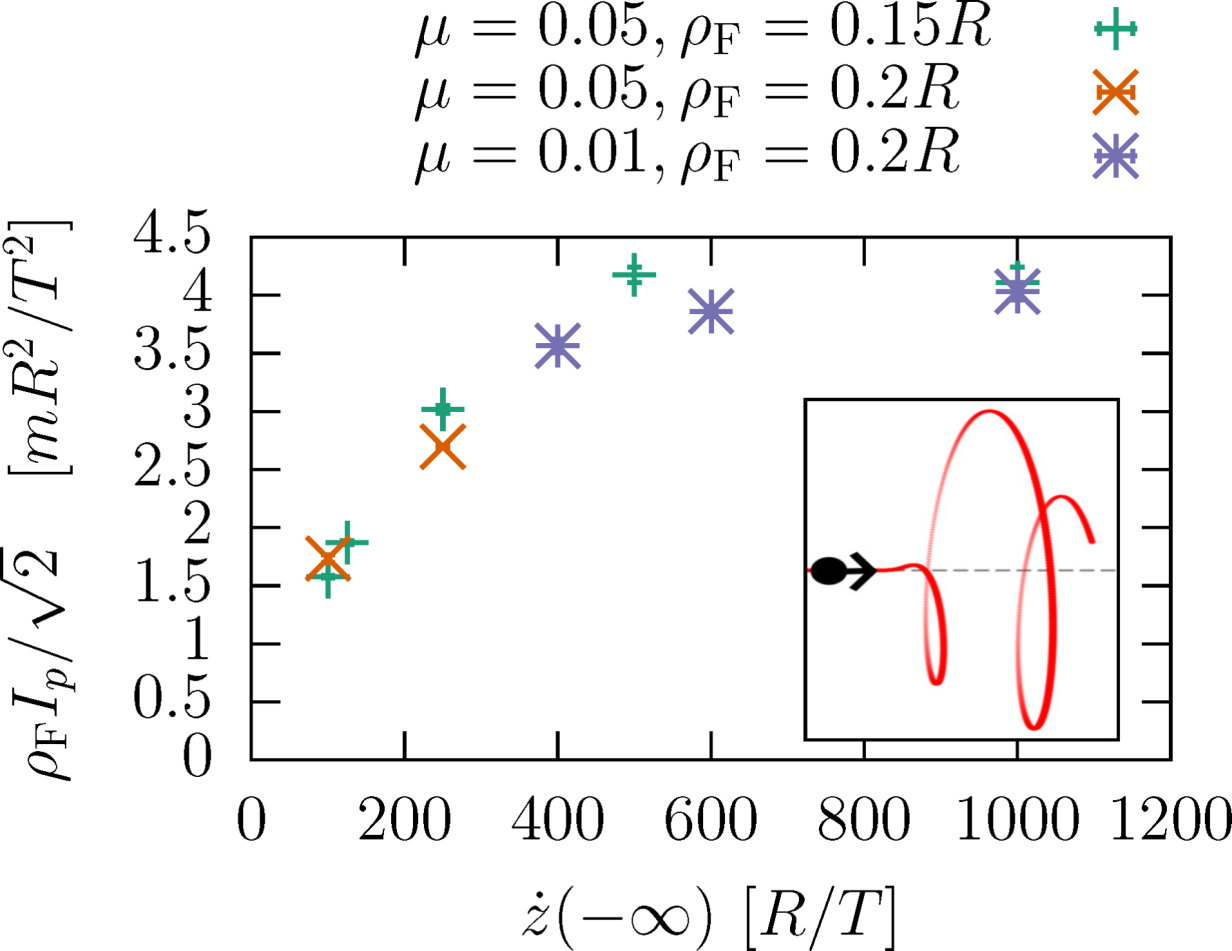
Velocity dependence of the threshold momentum current 
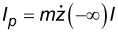
. The large-velocity limit is dominated by the garden hose effect, that is, angular momentum boosts.

To take a closer look at the mechanism of angular momentum boosts, we have plotted the threshold momentum current *I_p_* for different values of ρ_F_ and μ in Figure 7. The data collapses on a single curve, which saturates in the large impact velocity limit.

## Discussion

First, we discuss some straightforward extensions of our model that account for (1) friction and (2) temperature effects. Then, we discuss the prospective applications as a switch and for information storage. The role of quantum effects is discussed at the end.

Our model can be straightforwardly extended to include a friction term acting on the coordinate ϑ. Such friction can originate at the bearings of the rotor (at the entrance and exit of the path in our case). The projectile also experiences friction. Due to conservation laws, the loss of the angular momentum of the projectile is compensated by an increase of 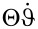. Therefore, this friction is included in our formulation.

Our results are valid when thermal fluctuations are small, that is, *k*_B_*T* ≪ Δ*V*. To account for fluctuations, it is customary to apply the Langevin equation for ϑ equipped with stochastic torques and a deterministic torque, the latter driving the directed rotation [11]. Our approach predicts the detailed form of the deterministic torque as it follows from the passage of the particle through a chiral path with ρ_F_ ∼ *R*. When ρ_F_ = 0, the effect of the passage (collision) is to boost the angle. In the stochastic equation, such a single-particle process can be accounted for by the torque of the form


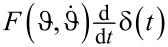


in the limit of short collision times. The derivative of a delta function expresses a torque pulse that is immediately cancelled by a pulse of opposite sign, thus generating no net 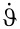 but a boost in ϑ. The function 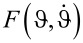 follows from our methodology straightforwardly.

We have focused largely on the conditions of a directed rotation. To implement an efficient switch, more conditions need to be fulfilled. First, the rotor’s velocity must be attenuated for the rotor to settle in the nearest potential minimum. Second, the current must flow in controlled short pulses. The optimal parameter regime can be sought using the EOM (Equation 20 and Equation 21), which is beyond the scope of the present work. We expect that the threshold current will vanish in a strongly overdamped limit and a linear response of 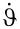 is expected.

The results presented here demonstrate a directed current-induced rotation without any angular momentum transfer in a molecular rotor. We remark that each electron boosts the angle of the rotor, but not its frequency. Hence, there is no net torque. Such devices can rotate under a particle current, but they cannot do work. Although they cannot operate as motors, these rotors can serve in nanoscale information storage and processing. The information readout can be performed in linear response (under the threshold current). A small symmetry breaking is needed in order to discriminate between the three states.

Quantum effects are responsible for a rich transport phenomenology of molecular junctions [20]. Here, we pause to discuss quantum effects related to the electronic degrees of freedom, assuming that the quantization levels of the rotational motion fall below the working temperature. Rotation only happens via inelastic electron tunneling. Importantly, each single electron scattering event must obey fundamental conservation laws. Therefore, the principles outlined in this manuscript will remain valid in the quantum limit. Two quantum aspects are significant in this context: (1) The electron transport process is stochastic, allowing for both transmission and reflection. Particle reflection off the helix can not induce any rotation, unless the following effect is considered. (2) Electrons carry spin angular momentum, which couples with the orbital momentum by spin–orbit interaction. Thus, reflection accompanied by a spin flip can induce angular momentum transfer.

## Conclusion

We have investigated the classical dynamics of a molecular rotor under a particle current. The molecule was modeled by a massive path that has a rotational degree of freedom. Our approach expresses the impact of a single collision on the rotor in a way that stems explicitly from the (chiral) geometry of the rotor.

When the particles do not carry (or take) any angular momentum, rotation is possible via angular boosts. If the rotation is hindered by a potential barrier Δ*V*, the requirement that the incident particles carry enough energy is not sufficient for switching. Instead, the stricter requirement that the boosts are sufficiently fast and dense in time applies (Equation 16).

When the particles are allowed to transfer angular momentum, we predict a crossover from the regime of angular boosts to the regime of angular momentum boosts.

## Appendix: Coordinates of an *N*-Helix

We introduce a path definition


[22]

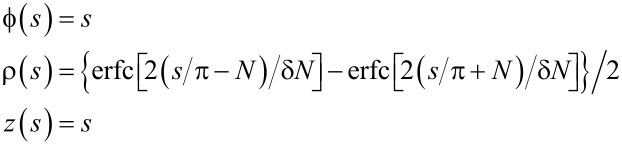



describing a helix with *N* turns, whose radius smoothly approaches zero at its both ends within a distance proportional to δ*N*, see Figure 1b. The smooth onset is achieved by employing the complementary error function, erfc. In the above definition, we adopt as a unit of length the maximum radius *R*.

This path will be employed for *s* ∈ (−∞, *s*_F_). When *s*_F_ = ∞, the condition in Equation 2 is fulfilled. Setting a finite *s*_F_ provides a path with an open end, when the particle exits the path at a finite ρ.

## Appendix: Equations of Motion

### EOM from the Lagrangian

The equations of motion (EOM) derived from a Lagrangian 
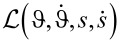
 by the principle of least action [21] read


[23]

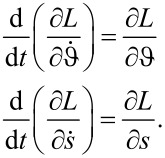



Inserting Equation 5, the EOM take the form


[20]

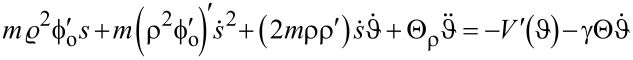




[21]

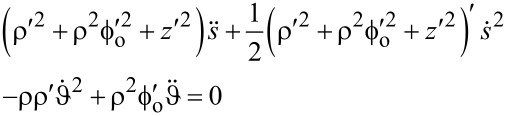



The first EOM delivers the equation for a rotor in the limit *m* = 0. We have added a phenomenological damping term 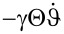 to the EOM (which does not follow from the conservative Lagrangian formalism). The damping term is zero in all numerical results of this work unless stated explicitly. When *V* = γ = 0, the equation expresses angular momentum conservation. The second equation describes the constrained particle dynamics if ϑ = const. Notice that the mass *m* vanishes, because the particle experiences inertial forces only.

### Transformation to dimension-less variables

#### EOM of the rotor

Substituting 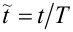 in Equation 20 renders the first EOM dimensionless,


[24]

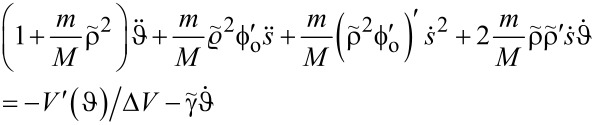



where now the dots indicate differentiation with respect to 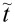 and we defined 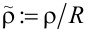, which is a quantity of the order of unity, and 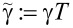 is a dimensionless damping rate. Notice the appearance of the small parameter 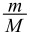, which, however, is here often multiplied by the large velocity 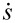.

#### EOM for the particle

The substitution of 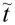 leaves the second EOM in the form


[25]

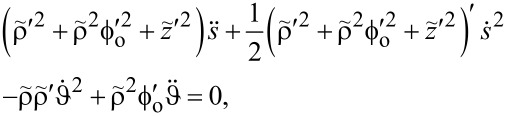



where we introduced 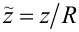 and the dot indicates differentiation with respect to 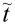. At the entry point, *s* = 0, the velocity of the particle equals d*z*(0)/d*t*. The expression 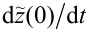 describes the inverse dwell time δτ. We shall assume that δτ = 10^−2^*T* and, hence, 
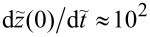
.

## Appendix: Peripheral numerical results

**Figure 8:**
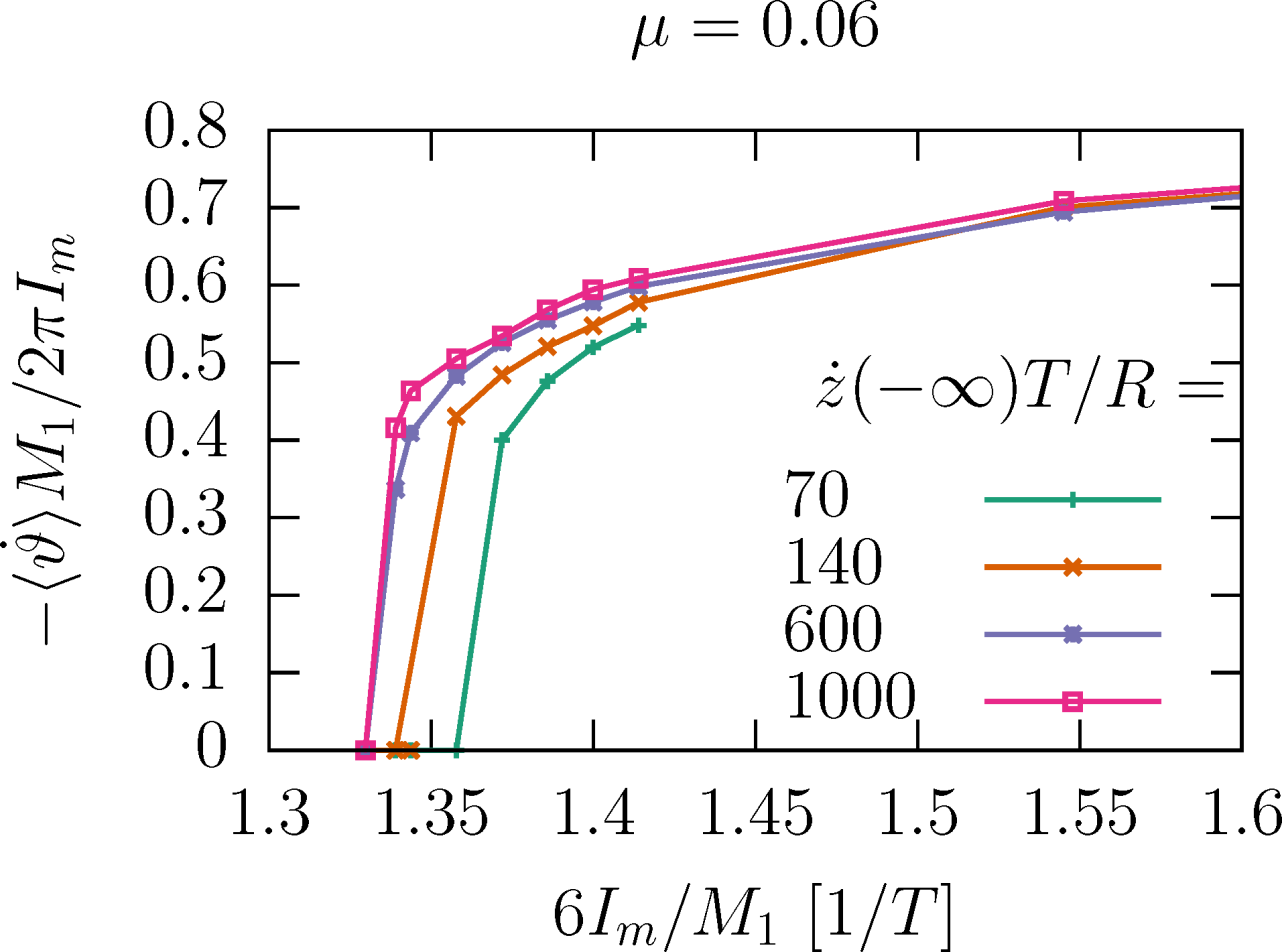
Velocity dependence of average angular velocity of the path exposed to a mass current *I_m_*. For lower velocities the threshold current increases, marking a departure from the SA.

**Figure 9:**
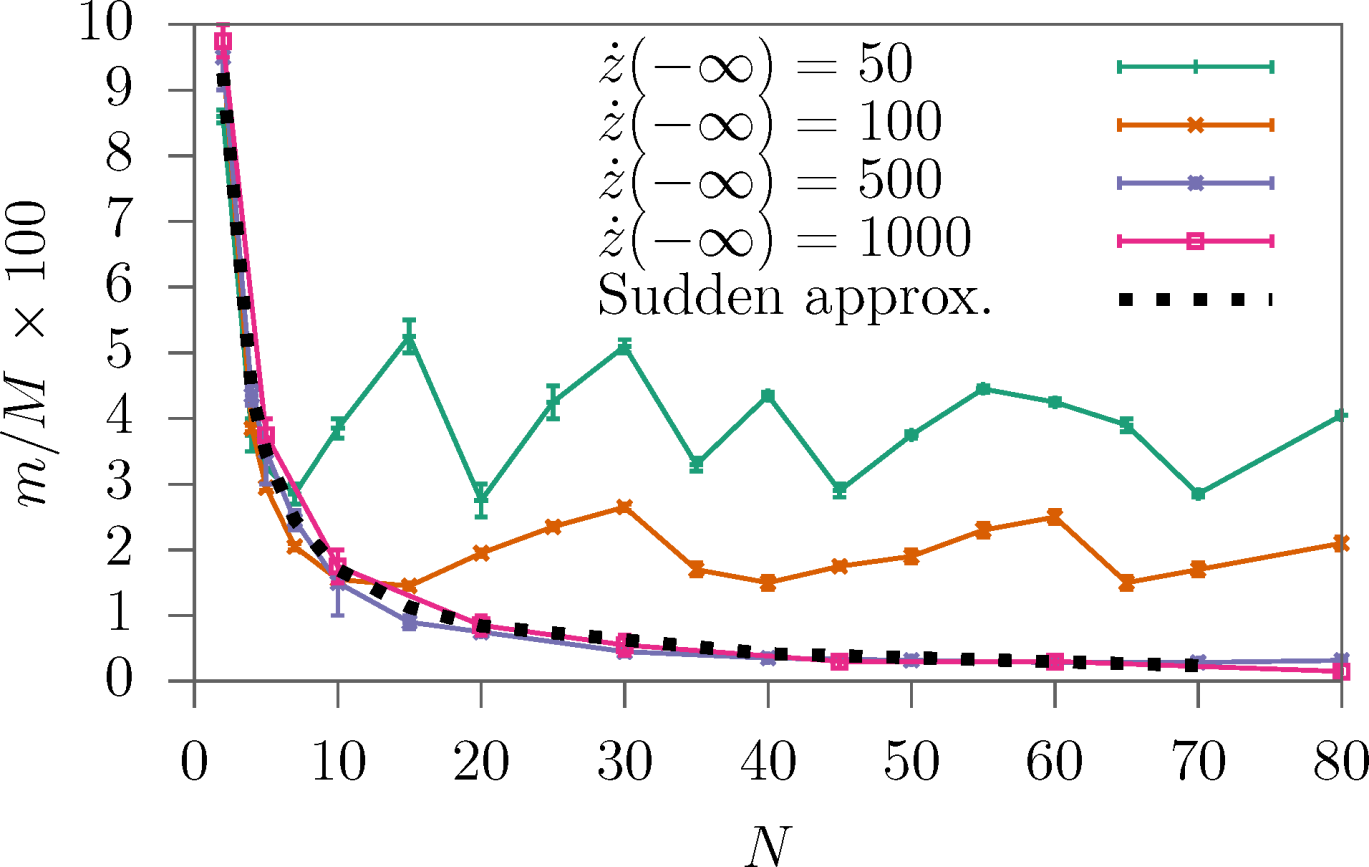
Threshold mass ratio *m*/*M* for an unbound directed motion as a function of the helix length *N* (δ*N* = 1) and the impact velocity. The dotted line is the threshold according to the SA, Equation 11, which coincides with the numerics if the time a particle spends in the helix is short.
